# Diacylglycerol Kinases: Shaping Diacylglycerol and Phosphatidic Acid Gradients to Control Cell Polarity

**DOI:** 10.3389/fcell.2016.00140

**Published:** 2016-11-29

**Authors:** Gianluca Baldanzi, Valentina Bettio, Valeria Malacarne, Andrea Graziani

**Affiliations:** ^1^Department of Translational Medicine, University of Piemonte OrientaleNovara, Italy; ^2^Institute for Research and Cure of Autoimmune DiseasesNovara, Italy; ^3^Division of Experimental Oncology, School of Medicine, University Vita e Salute San RaffaeleMilan, Italy

**Keywords:** immune synapse, migration, lipid domain, cell polarity, localization

## Abstract

Diacylglycerol kinases (DGKs) terminate diacylglycerol (DAG) signaling and promote phosphatidic acid (PA) production. Isoform specific regulation of DGKs activity and localization allows DGKs to shape the DAG and PA gradients. The capacity of DGKs to constrain the areas of DAG signaling is exemplified by their role in defining the contact interface between T cells and antigen presenting cells: the immune synapse. Upon T cell receptor engagement, both DGK α and ζ metabolize DAG at the immune synapse thus constraining DAG signaling. Interestingly, their activity and localization are not fully redundant because DGKζ activity metabolizes the bulk of DAG in the cell, whereas DGKα limits the DAG signaling area localizing specifically at the periphery of the immune synapse. When DGKs terminate DAG signaling, the local PA production defines a new signaling domain, where PA recruits and activates a second wave of effector proteins. The best-characterized example is the role of DGKs in protrusion elongation and cell migration. Indeed, upon growth factor stimulation, several DGK isoforms, such as α, ζ, and γ, are recruited and activated at the plasma membrane. Here, local PA production controls cell migration by finely modulating cytoskeletal remodeling and integrin recycling. Interestingly, DGK-produced PA also controls the localization and activity of key players in cell polarity such as aPKC, Par3, and integrin β1. Thus, T cell polarization and directional migration may be just two instances of the general contribution of DGKs to the definition of cell polarity by local specification of membrane identity signaling.

## Membrane identity and cell polarity

The compartmentalization of plasma membrane proteins is a common characteristic of eukaryotic cells and provides the base for the establishment of signaling domains (Spira et al., 2012). Local changes in lipid distribution also contribute to plasma membrane heterogeneity. However, the actual size and stability of lipid domains in cell membranes is still debatable (Carquin et al., 2016). The presence of specific lipids in different cellular compartments, including the plasma membrane, is required for the localized recruitment of effector proteins like actin regulators, protein kinases, and small GTPases (Lemmon, 2008).

The contribution of phosphoinositides (PIs) to cell organization has been extensively characterized; specific PIs play a major role in determining the subcellular identity of membranes (Di Paolo and De Camilli, 2006). The balance between Phosphatidylinositol-trisphosphate (PI_3,4,5_P3) (Di Paolo and De Camilli, 2006; Lemmon, 2008; Sánchez-Madrid and Serrador, 2009) and Phosphatidylinositol-bisphosphate (PI_4,5_P2) (Di Paolo and De Camilli, 2006; Sánchez-Madrid and Serrador, 2009) is specifically involved in the generation and maintenance of cell polarity in a variety of experimental systems. In migrating leukocytes as well as in Dictyostelium cells, a PI_3,4,5_P3 → PI_4,5_P2 gradient identifies the leading edge of the cell compared to the uropod at the rear (Sánchez-Madrid and Serrador, 2009). Similarly, in neurons, PI_3,4,5_P3 is enriched at the tip of the growing axon (Shi et al., 2003). In apical/basal polarized epithelial cells, PI_4,5_P2 accumulates at the apical domain whereas the basolateral membranes are enriched in PI_3,4,5_P3 (Gassama-Diagne et al., 2006). In all these cases, local lipid enrichment results from the spatial segregation of PI_3,4,5_P3 generation by PI3Ks and its metabolism by PTEN activity (Funamoto et al., 2002; Lacalle et al., 2004; Martin-Belmonte et al., 2007; Leslie et al., 2008). Once established, the uneven distribution of PIs promotes cell polarization by recruiting specific effectors.

Similar to PI_3,4,5_P3 and PI_4,5_P2, both DAG and PA are: (i) second messengers that recruit a set of interacting proteins (Mérida et al., 2008), (ii) kept in balance by the combined action of PA phosphatases and DGKs (Sakane et al., 2007) and (iii) enriched in specific domains of the plasma membrane where they recruit specific effectors.

In quiescent cells grown on bi-dimensional surfaces, PA is present in relevant amounts with prominent distribution at the free edges compared with that at cell-cell contacts (Nishioka et al., 2010). Further PA production by PLD and DGKs is observed upon receptor triggering, with highest levels at the nascent lamellipodia (Nishioka et al., 2010). Herein, PA binding proteins such as aPKC (Chianale et al., 2010; Rainero et al., 2014) or Nir2 (Kim et al., 2013), drive cytoskeletal remodeling and protrusion elongation. Similarly, PA participates in the recruitment of the Rac activator, DOCK1, during dorsal ruffle formation in fibroblasts (Sanematsu et al., 2013), as well as in the localization and activation of the Rac-RhoGAP complex during neurite outgrowth (Kurooka et al., 2011). Moreover, DAG is locally produced by the activity of extracellular regulated phospholipase C (PLC) on PI_4,5_P2 (Kadamur and Ross, 2013) and by the PA phosphatase activity at both the plasma membrane and in the intracellular organelles (Brindley et al., 2009). The resulting DAG production is essential to many biological systems such as the immune synapse, the neuronal synapse, and phagocytosis (Almena and Mérida, 2011).

PA and DAG gradients are somehow different in migrating cells. Indeed, low PA levels have been found at the leading edge of spontaneously migrating HeLa cells compared to those at the trailing edge (Ferraz-Nogueira et al., 2014), whereas DAG is symmetrically enriched at the front of migrating cells (Nishioka et al., 2008). Similarly, PA is strongly decreased at the apical domain of polarized epithelial cells, whereas DAG is lightly enriched (Gerl et al., 2012). These data demonstrate the existence of PA and DAG enriched domains that contribute to cellular asymmetry and thus suggest a putative role of DGKs in the control of cell polarity. Accordingly, while in E. coli DGK is a transmembrane protein that phosphorylates multiple lipids (Van Horn and Sanders, 2012), mammalian DGKs are soluble enzymes recruited on demand at specific cellular locations where they act on specific DAG pools (Kobayashi et al., 2007). The relevance of targeting to specific membrane domains is evidenced by the presence of multiple domains controlling membrane association, in the N terminal part of all DGKs, apart from DGKε (Mérida et al., 2008).

Here, we will discuss some well-characterized examples of the contribution of DGK activity to the generation and maintenance of lipid signaling domains in polarized cells.

## DGKα and ζ at the immunological synapse

The contact zone between the T cell and the antigen-presenting cell (APC) is a specialized structure described as the immunological synapse (IS) (Monks et al., 1998; Grakoui et al., 1999). The IS has a well-defined spatial organization where supramolecular activation clusters (SMACs) are arranged in radial symmetry to form a “bull's eye” shape (Monks et al., 1998). The more distal zone (dSMAC) is CD45-enriched and is characterized by active actin movements resembling the sensory lamellipodia of epithelial cells (Dustin et al., 2010). This is followed by a peripheral zone (pSMAC) enriched in adhesion molecules such as LFA-1 (lymphocyte function-associated antigen-1, integrin αLβ2), and VLA4 (Very Late Antigen-4, integrin α4β1) and the associated talin that resemble adhesive lamella (Mittelbrunn et al., 2004). In the central part (cSMAC), coactivators (e.g., CD28) and kinases (LCK, Fyn) are enriched, but endocytosis also occurs, resembling that in uropods of migrating cells. The cSMAC is also the site of secretion of cytokines, cytolytic agents, and exosomes into the synapse (Dustin, 2014).

Upon antigen stimulation, T cell receptor (TCR) microclusters form at the IS periphery and move toward the cSMAC where they encounter the endocytic sorting machinery and are internalized. IS formation drives the polarization of the entire T cell, with the translocation of the microtubule organizing center (MTOC) between the IS and the nucleus, and the establishment of the uropod, a membrane zone enriched in signaling molecules at the opposite end of the T cell (Serrador et al., 1999). Both IS formation and repositioning of the MTOC are key events during the killing of a cognate target cell by cytotoxic T lymphocytes (CTLs). Cytotoxic granules move along microtubules and the granule content is released between the CTL and the target cell, where perforin and granzymes co-operate to induce rapid death of the target cell by apoptosis (de Saint Basile et al., 2010).

Unstimulated T cells display uniform distribution of DAG at the plasma membrane, whereas after T cell activation, a DAG gradient is established at the center of the IS by the activity of TCR-activated PLCγ (Spitaler et al., 2006) and by the combined action of PLD and PA-phosphatases (Mor et al., 2007). This DAG is essential for the recruitment of downstream DAG-dependent effectors such as conventional PKC (cPKCs), PKD, and RasGRP1, which promote the downstream T cell responses (Spitaler et al., 2006). As recently reviewed by Merida et al., rapid DAG metabolism occurs at the IS (Mérida et al., 2015). Indeed both DGKα and DGKζ are translocated to the proximal and distal poles of the T cell during IS formation (Joshi et al., 2013) and both DGKα and DGKζ are recruited to the TCR complex (Gerl et al., 2012). Despite a substantial overlap in localization upon TCR triggering and their common function as negative regulators of TCR-downstream signaling (Zhong et al., 2003; Olenchock et al., 2006), the roles of DGKα and DGKζ do not seem fully redundant. Indeed, TCR triggers DGKζ phosphorylation on the myristoylated alanine-rich C-kinase substrate (MARKS) domain by PKC (Gharbi et al., 2011). Upon phosphorylation, DGKζ spreads among the entire immunological synapse, where it contributes to DAG metabolism (Gharbi et al., 2011; Joshi et al., 2013). Conversely, DGKα is selectively recruited to the periphery of the IS in a PI3Kδ dependent manner (Chauveau et al., 2014). Membrane-associated DGKα is phosphorylated on Y_335_ and is activated by Lck and Ca^2+^ with a timing that overlaps with PLCγ-phosphorylation (Sanjuán et al., 2001; Merino et al., 2008). *In vitro* experiments show that membrane-localized DGKα in T cells displays a substantial overlap with the F-actin ring surrounding the central DAG bulk, where DGKα plays a specific role in restricting the DAG domain. Indeed, in WT and DGKζ^−/−^ T cells, the DAG probe C1δ-GFP was localized within this F-actin ring, whereas in DGKα^−/−^ cells, DAG distribution appeared substantially broader (Chauveau et al., 2014). Thus, DGKα contributes to polarity determination by constraining DAG accumulation into the cSMAC, while DGKζ plays a general function in reducing the intensity of TCR-downstream signaling (Chauveau et al., 2014).

The DGKα-mediated shaping of DAG gradient at the immune synapse is required for T cell polarization as DGKα^−/−^ cells show partial impairment in TCR-promoted MTOC re-localization and polarized secretion (Quann et al., 2009; Alonso et al., 2011; Chauveau et al., 2014). Absence of DGK activity closely resembles T cell treatment with DAG analogs, such as phorbol esters, which completely abrogate MTOC reorientation toward the IS (Quann et al., 2009). The relevance of DGKα in T cell polarization is less evident when assayed in conjugates between antigen presenting B cells and Jurkat T cells. In these IS, DGKα inhibition does not perturb MTOC and F-actin polarization, but significantly affects DAG accumulation at the IS, suggesting that some polarization events also occur in the absence of localized DAG signaling (Ruffo et al., 2016). A striking example of the functional relevance of DGKα in the control of T cell polarization is the X-linked lymphoproliferative disease 1 (XLP-1). XLP-1 is a primary immunodeficiency due to defects in signaling lymphocytic activation molecule (SLAM)–associated protein (SAP), an adaptor protein that modulates TCR-induced signaling. We have demonstrated that the SLAM-SAP signaling axis negatively regulates DGKα activity in T cells (Baldanzi et al., 2011a). In XLP-1 SAP is mutated or absent and results in constitutive DGKα activity that blunts the DAG dependent TCR signaling (Dustin et al., 2010). Interestingly, SAP deficient cells not only show partial impairment of TCR signaling, but also have specific defects in novel PKC (nPKC) recruitment at the IS (Cannons et al., 2004, 2010a), thus reducing the IS stability ad effector functions (Cannons et al., 2010b; Zhao et al., 2012). These defects are due to excessive DGKα activity, as silencing or inhibiting DGKα in SAP deficient cells restores the correct level of DAG and its effectors at the IS and reestablishes MTOC polarization (Ruffo et al., 2016). Accordingly, inhibition of DGKα activity had no substantial effect on the killing of target cells by activated CD8^+^ lymphocytes whereas it enhances the weak effector function of SAP deficient lymphocytes (Chauveau et al., 2014; Ruffo et al., 2016).

**Figure 1:**
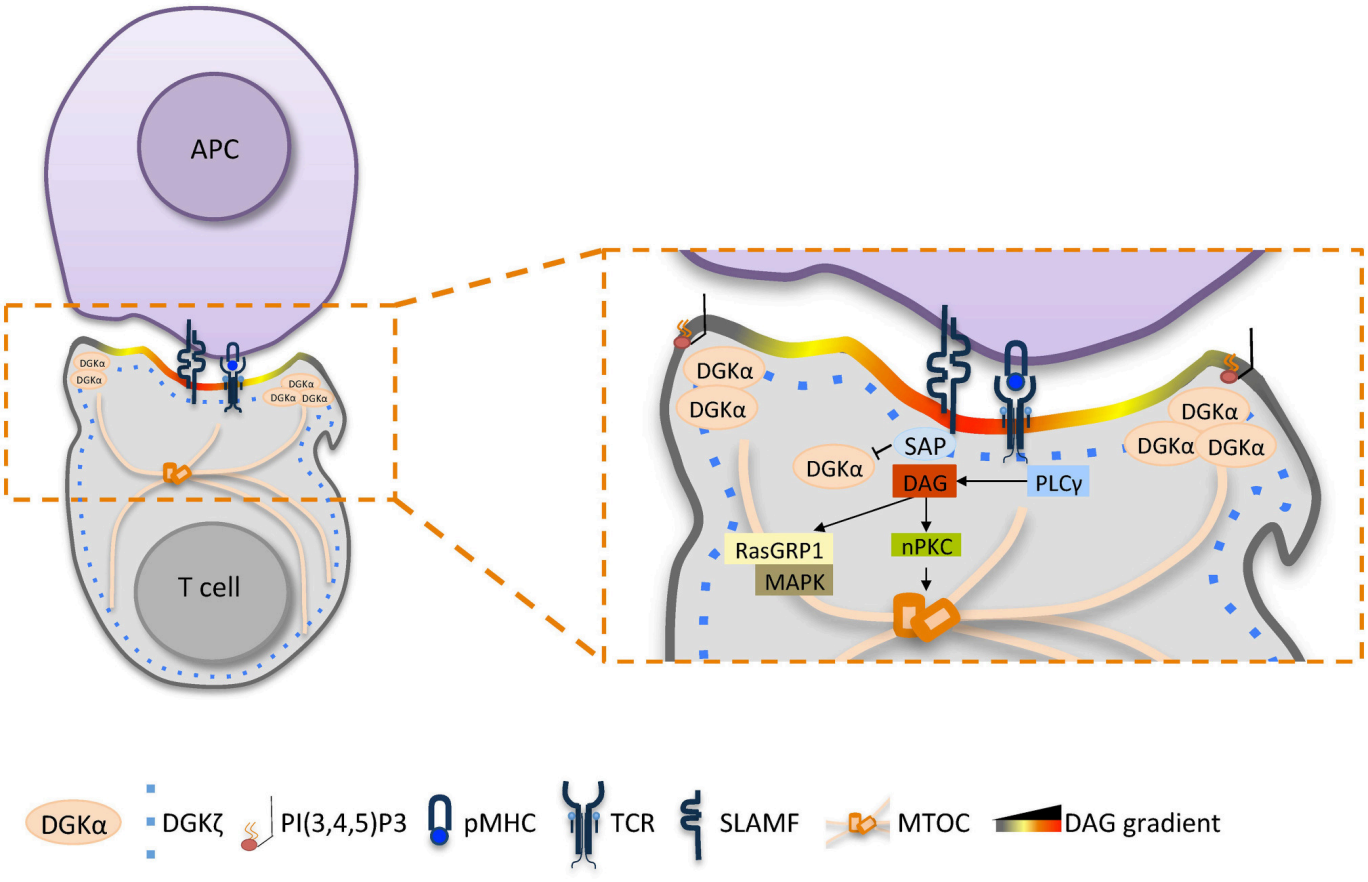
**The role of DGKα in shaping diacylglycerol signaling at the immune synapse**. Upon TCR/SLAM engagement, PLCγ activation induces DAG production at the synapse and DGKα/ζ recruitment. DGKζ (blue dotted line) is responsible for the metabolism of the bulk of DAG over the entire synapse, whereas DGKα (light orange) is recruited by PI3Kδ generated PI_3,4,5_P3 at the pSMAC, where DAG is constrained. Excessive DAG metabolism at the IS by DGKα is prevented by SAP-mediated inhibition. The resulting sharp DAG gradient promotes downstream MAPK signaling and the local recruitment of nPKC, which promotes MTOC reorientation.

These observations indicate that a signaling domain enriched in DAG is generated by the fine-tuning of localized production by PLCγ and equally localized metabolism by DGKα (Mérida et al., 2015). As evidenced in Figure 1, fine regulation of DGKα activity plays a central role, with a small fraction recruited and activated by PI3Kδ at the pSMAC (Chauveau et al., 2014), and the remaining enzyme activity is inhibited by SAP (Baldanzi et al., 2011a). The resulting spatial definition of DAG signaling drives T cell polarization by promoting the local recruitment of multiple PKC isoforms (Quann et al., 2011) that in turn organize molecular motors such as dynein at the IS and myosin II at the opposite cell end (Liu et al., 2013). Once established, T cell polarity is maintained by polarized vesicular trafficking toward the IS, where DGKα also plays a role. Indeed, DGKα is recruited to multivesicular bodies and to exosomes, and it promotes both the polarization of MVBs toward the IS and exosome secretion (Alonso et al., 2011).

Little is known about the function of DGKs in other types of immune synapses. In NK cells, DGKζ silencing or treatment with DGK inhibitors enhances effector functions (Prinz et al., 2014; Yang et al., 2016), but it is currently unknown if this relates to a change in the DAG gradient or to an increased efficiency of the secretory pathways.

Notably, while the role of DGKα as a DAG-driven signaling terminator has been extensively investigated, whether and how PA production by DGKs at the IS affects TCR signaling is currently unknown. However, some cues suggest this possibility as PA production by DGKα and ζ is required for T cell development (Guo et al., 2008), whereas DGKζ-generated PA promotes TLR-induced IL-12 production by negatively regulating the PI3K-AKT pathway (Liu et al., 2007). Moreover, PA is an allosteric activator of PLCγ (Jones and Carpenter, 1993), suggesting that a PA-dependent feedback mechanism can amplify the magnitude of the signal. Future studies addressing the localization and function of DGKs-generated PA in lymphocytes should provide further insights into TCR signaling.

## DGKs in directional migration

**Figure 2:**
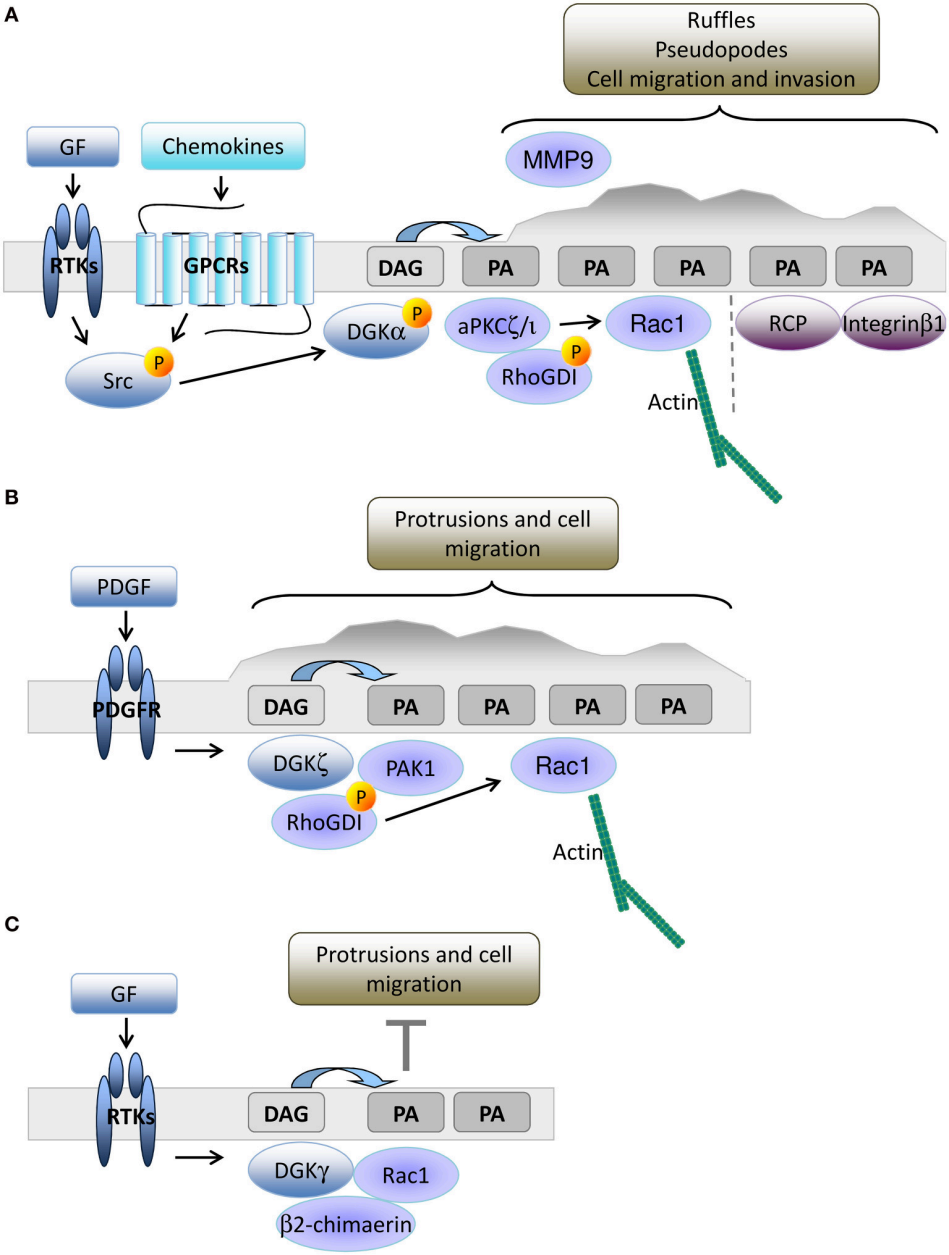
**DGKs-produced PA drives membrane protrusion and cell motility. (A)** In epithelial cells, growth factors or chemokine stimulation promotes Src mediated recruitment and activation of DGKα to nascent protrusions. The resulting PA enriched domain drives protrusion elongation and matrix invasion by the local recruitment of aPKC and Rab11-FIP1. These PA binding proteins respectively control cytoskeletal remodeling through Rac1 and integrin β1 recycling. **(B)** In fibroblasts, PDGF triggers DGKζ recruitment to nascent protrusions. The resulting PA enriched domain drives focal adhesion remodeling and cell migration by recruiting and activating PAK1 and promoting Rac1 activation. **(C)** Growth factors also promote membrane recruitment and activation of DGKγ that limits Rac1 activity through β2 chimaerin.

The leading edge of growth factor stimulated cells is another site of intense PIPs turnover, where PLCγ is recruited to produce a local enrichment of DAG coupled to Ca^2+^ release triggered by inositol triphosphate (Piccolo et al., 2002; Mouneimne et al., 2004; Nishioka et al., 2008). We have demonstrated that upon growth factor or chemokine-mediated stimulation of epithelial cells, DGKα is activated and recruited to the plasma membrane, where the PA produced by DGKα recruits PA-binding proteins such as atypical PKC ζ and (aPKCζ; Chianale et al., 2007, 2010; Baldanzi et al., 2008) and Rab11 family interacting protein 1 (Rab11-FIP1) (Rainero et al., 2012). DGKα activated aPKC phosphorylates RhoGDI, thus promoting the release of Rac1, actin polymerization, and elongation of invasive protrusions enriched in Integrin β_1_ and metalloproteinase 9 (Chianale et al., 2007, 2010; Rainero et al., 2014). The PA produced by DGKα at the tip of invasive pseudopods is also a docking site for vesicles containing Rab11-FIP1 and Integrin α_5_β_1_, allowing DGKα to polarize vesicular trafficking and promote directional migration (Rainero et al., 2014). Altogether, these data indicate that in epithelial cells, PA production by DGKα is essential to orchestrate the organization of the signaling machinery that promotes protrusion formation and directed cell migration (Figure 2A).

DGKα expression is low in mouse embryonic fibroblasts (MEFs), but other investigators have demonstrated that DGKζ plays an equivalent role in cell migration. DGKζ^−/−^ MEFs have more focal adhesions at their central region due to impairment in the local recruitment of PAK1 kinase. Upon PDGF stimulation, DGKζ promotes PAK1-mediated phosphorylation of RhoGDI that releases Rac1, which drives directed cell migration (Abramovici et al., 2009). Thus, close parallelism exists between the DGKα driven aPKC recruitment in epithelial cells and the DGKζ mediated recruitment of PAK1 in MEFs, both controlling Rac1 activity and migration. Moreover, DGKζ also regulates RhoA activity in MEFs by acting as a scaffolding protein independently of PA production (Ard et al., 2012). DGKζ is also highly expressed in colon cancer cell lines and its expression correlates with enhanced cell motility due to increased Rac1 and RhoA activation (Cai et al., 2014), suggesting that DGKζ is a key regulator of Rho GTPase activity and cell migration in fibroblasts and tumor cells (Figure 2B).

In fibroblasts, DGKγ is also recruited to ruffles and lamellipodia where it co-localizes with Rac1 (Figure 2C). Surprisingly, DGKγ acts as a suppressor of growth factor-induced protrusions by recruiting and activating the β2-chimerin GAP activity (Tsushima et al., 2004; Yasuda et al., 2007). This observation clearly indicates the key contribution of DGKs to local PA accumulation that controls ruffling and lamellipodia formation (Nishioka et al., 2010), but suggests that multiple PA pools with specific functions and interactors are involved. The relevance of protein-protein interactions in dictating the signaling outcome of DGK-produced PA is evidenced by the observation of the isoform specific complexes (i) DGKα-aPKC-RhoGDI-Rac1 (Chianale et al., 2010), (ii) DGKζ-PAK1-RhoGDI-Rac1 (Abramovici et al., 2009), and (iii) DGKγ-β2 chimaerin (Yasuda et al., 2007).

The relevance of DAG and PA in cell migration suggests that DGKs are relevant targets for the control of tumor development and metastasis (Purow, 2015). Surprisingly, both DGKα and ζ also play a key role in cancer-cell survival, acting at either the plasma membrane, intracellular organelles, or the nucleus (Baldanzi et al., 2011b; Filigheddu et al., 2011; Dominguez et al., 2013; Kefas et al., 2013; Tanaka et al., 2013; Torres-Ayuso et al., 2014, 2015; Poli et al., 2016). However, the signaling pathways that converts PA and DAG at cell membranes in cell survival signaling are poorly understood, as few PA-regulated proteins are linked to the control of apoptosis and the cell cycle. Among these, mTOR (mammalian target of rapamycin) is notable as a target for both PI_3,4,5_P3 signaling and DGKζ-produced PA (Avila-Flores et al., 2005; Chen et al., 2012; You et al., 2014).

## Future prospects: do DGKs play a role in the control of cell polarity?

The illustrated data indicate the role of DGKs in the shaping of signaling domains at the plasma membrane by confining DAG signaling and contributing to the generation of PA-enriched domains. In the T cells engaged in IS, the existence of a DAG gradient is necessary and sufficient to polarize the entire cytoskeleton (Quann et al., 2009) but the relevance of DAG gradients in other polarized systems such as front/rear or apical/basal asymmetry is currently under investigation (Tsai et al., 2014).

PA gradients are even less characterized because the relevance of PA in signaling is just emerging (Jang et al., 2012). A few studies found a role of PLD generated PA in the recruitment of PA binding proteins to the apical domain of epithelial monolayers (Gloerich et al., 2012; Consonni et al., 2014), despite controversial evidences about the apical enrichment of PA (Gerl et al., 2012). Interestingly, several data link the diacylglycerol generated-PA with central players in the establishment of cell polarity: aPKC, Par3, and integrin β1 (Rainero et al., 2014). In apical/basal polarized epithelial cells, aPKC, Par3, and Par6 compose the Par complex, which is located at the apical side within the region of tight junctions, where it promotes the formation and maintenance of tight junctions and the apical domain (Horikoshi et al., 2009). Activation of aPKC is a key event in the regulation of apical/basal polarity since aPKC phosphorylates several substrates involved in polarity establishment such as Crumbs, Lgl, and GSK3β (glycogen synthase kinase-3β). Phosphorylation of Crumbs and Lgl promotes their correct intracellular localization, whereas GSK3β phosphorylation is involved in microtubule capture and stabilization, and in the maturation of cell-cell contacts (Gandalovičová et al., 2016). The link with DGKs is provided by the observation that: i) aPKC binds to and is activated by PA (Limatola et al., 1994) and ii) their localization at the invasive protrusions of cancer cells is promoted by DGKα-produced PA (Chianale et al., 2010; Rainero et al., 2014). PA might also play a role in the localization of the aPKC-Par3-Par6 complex as the *Drosophila* Par3 homolog, Bazooka also directly binds PA (Yu and Harris, 2012).

DGKα-produced PA also controls intracellular trafficking of integrin β1 through the PA binding protein Rab11-FIP1 (Lindsay and McCaffrey, 2004; Rainero et al., 2012). Interestingly, integrin β1 trafficking is essential for directional migration (Shafaq-Zadah et al., 2016), apical/basal polarity (Bryant et al., 2014), and mitotic spindle orientation (Toyoshima and Nishida, 2007). Several evidences link integrin signaling and epithelial cell polarity (Zovein et al., 2010; Myllymäki et al., 2011), suggesting an interplay between integrin trafficking and Par complex activity. We speculate that DGKs-produced PA, which regulates both the Par complex through aPKC and integrin β1 trafficking though Rab11-FIP1, contributes to the coordination of those pathways.

Starting from the observation of the central role of DGKs in establishing lymphocyte polarity and in directional migration, we propose that this family of enzymes may play a widespread role in the establishment of membrane domains that dictates cell polarization. The neuronal system is a very promising field to explore, where several DGK isoforms are expressed (Ishisaka and Hara, 2014) and control both neurite growth and branching (Shirai et al., 2010) and synapse stability (Kim et al., 2009; Shirai et al., 2010).

This review aims to prompt further studies investigating the link between DGKs activity, membrane asymmetry, and cell fate.

## Author contributions

All authors listed, have made substantial, direct and intellectual contribution to the work, and approved it for publication.

## Funding

AG is supported by grants from Telethon (GGP13254), AIRC (Associazione Italiana per la Ricerca sul Cancro) (IG13524) and PRIN (2015 4CWJH4). GB is supported by the TIPSO grant from Regione Piemonte (PAR FSC 2007–2013 Asse I—Innovazione e transizione produttiva—Linea di azione 3: “Competitività industria e artigianato” linea d—Bando regionale sullemalattie Autoimmuni e Allergiche). Salary of VM and VB is provided by Telethon and TIPSO to AG and GB respectively.

### Conflict of interest statement

The authors declare that the research was conducted in the absence of any commercial or financial relationships that could be construed as a potential conflict of interest.
